# Supplementary material for: Luminescence properties of Ge and Ge-Si structures on silicon-based microcavities
Source: PLoS One. 2025 May 5;20(5):e0322559. doi: 10.1371/journal.pone.0322559 (PMC12052192; doi:10.1371/journal.pone.0322559)
Supplement: S1 File — S2 Fig. Characterization of the microcavity germanium-coated samples: (a) Secondary electron scanning electron microscope image at the edge of the microcavity, (b) enlarged view of part (a), (c) EDS spectrum at the edge of the microcavity. S3 Fig. Characterization of the germanium-coated microcavity samples: (a) XRD pattern of the sample, (b) photoluminescence spectroscopy of the sample. S4 Fig. XRD patterns of the germanium-coated microcavity samples at different annealing times: (a) after annealing for 10 min, (b) after annealing for 20 min, (c) after annealing for 30 min, (d) after annealing for 40 min, (e) after annealing for 50 min, (f) after annealing for 60 min. S5 Fig. Photoluminescence spectroscopy of germanium-coated microcavity samples at different annealing times: (a) after annealing for 10 min, (b) after annealing for 20 min, (c) after annealing for 30 min, (d) after annealing for 40 min, (e) after annealing for 50 min, and (f) after annealing for 60 min. S6 Fig. Characterization of the germanium-coated microcavity sample annealed for 40 min: (a) SEM image and (b) EDS spectrum. S7 Fig. Characterization of the Ge-Si coated microcavity samples: (a) the SEM image at the edge of the microcavity, (b) an enlarged view of (a), and (c) the EDS spectrum at the edge of the microcavity. S8 Fig. Characterization of the Ge-Si coated microcavity samples: (a) the XRD patterns, and (b) the photoluminescence spectroscopy of the samples. S9 Fig.XRD patterns of Ge-Si coated microcavity samples after different annealing times: (a) XRD patterns after 10 minutes of annealing, (b) XRD patterns after 20 minutes of annealing, (c) XRD patterns after 30 minutes of annealing, (d) XRD patterns after 40 minutes of annealing, (e) XRD patterns after 50 minutes of annealing, (f) XRD patterns after 60 minutes of annealing. S10 Fig. Photoluminescence spectroscopy of Ge-Si Microcavity Samples at Different Annealing Times: (a) the spectrum after 10 min; (b) after 20 min; (c) after 30 min; [file pone.0322559.s001.docx]

## **Supporting Information**


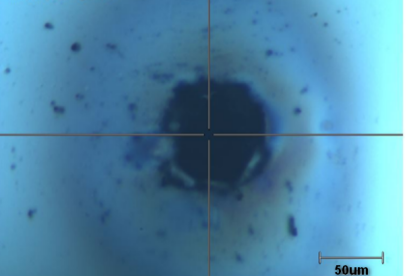

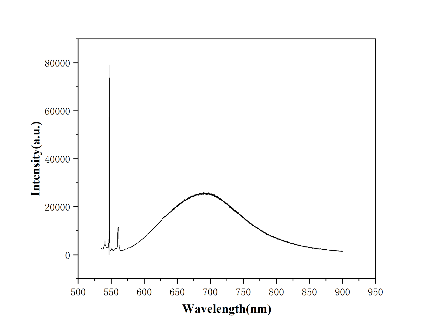


1. (b)

**S1 Fig. Characterization of the microcavity samples: (a) a microscopic image of the microcavity; (b) the photoluminescence spectroscopy of the sample.**


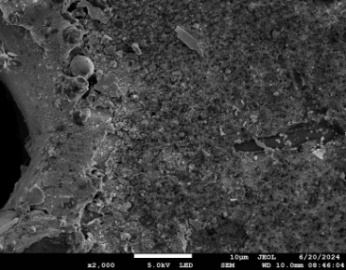

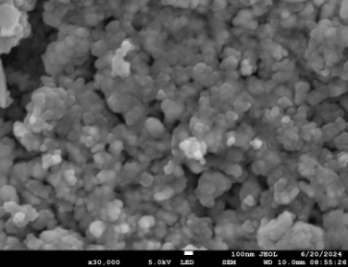


1. (b)


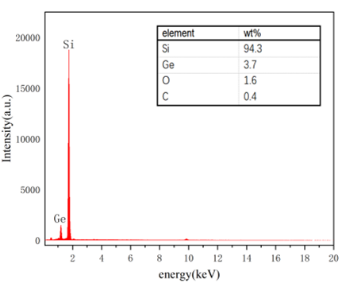


(c)

**S2 Fig. Characterization of the microcavity germanium-coated samples: (a) Secondary electron scanning electron microscope image at the edge of the microcavity, (b) enlarged view of part (a), (c) EDS spectrum at the edge of the microcavity.**


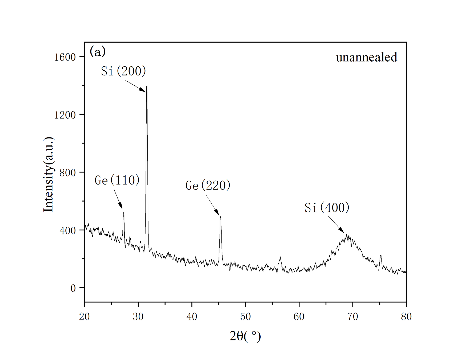

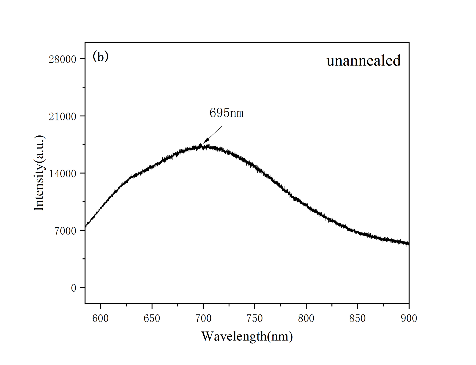


**S3 Fig. Characterization of the germanium-coated microcavity samples: (a) XRD pattern of the sample, (b) photoluminescence spectroscopy of the sample.**


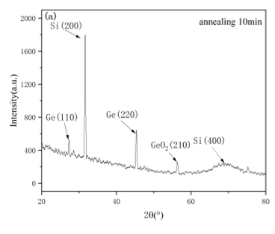

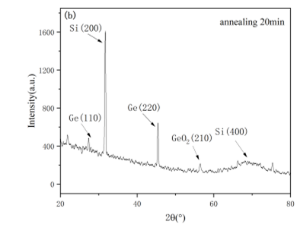

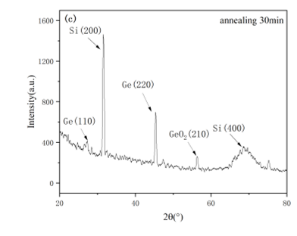

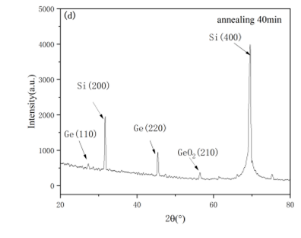

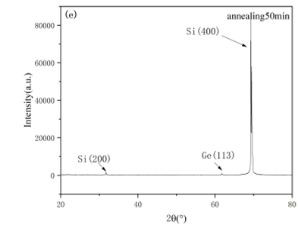

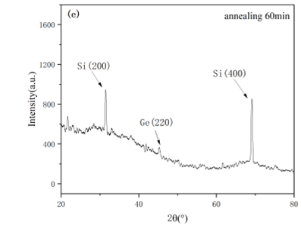


**S4 Fig. XRD patterns of the germanium-coated microcavity samples at different annealing times: (a) after annealing for 10 min, (b) after annealing for 20 min, (c) after annealing for 30 min, (d) after annealing for 40 min, (e) after annealing for 50 min, (f) after annealing for 60 min.**


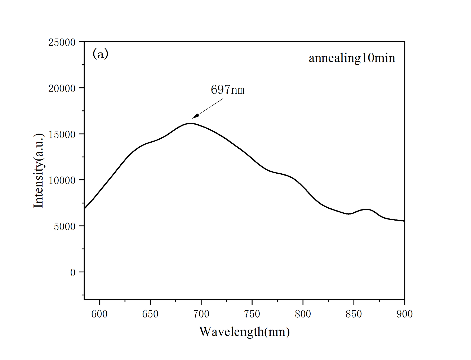

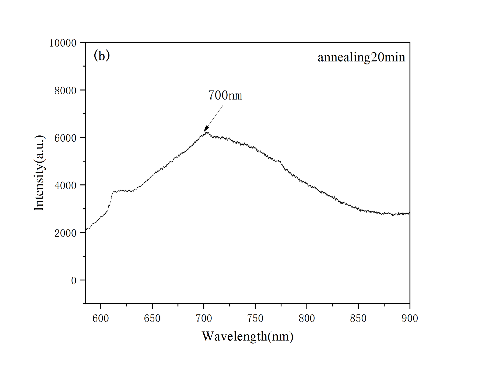

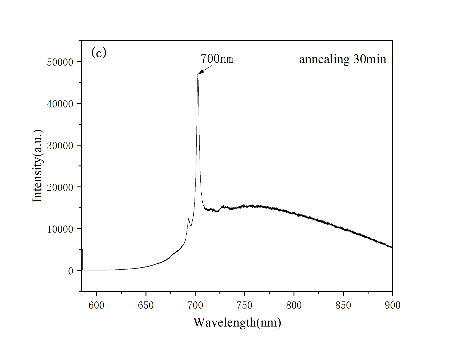

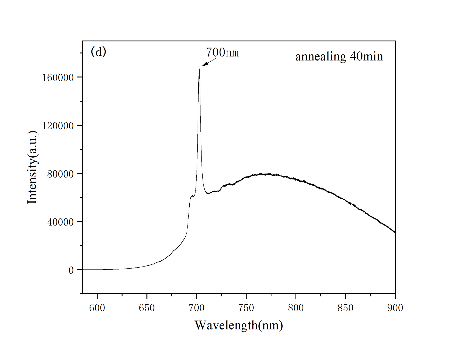

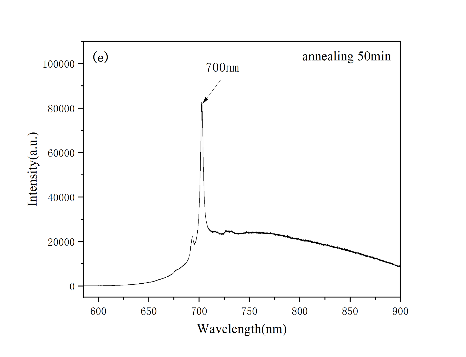

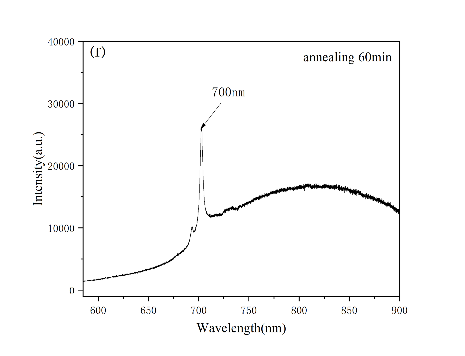


**S5 Fig. Photoluminescence spectroscopy of germanium-coated microcavity samples at different annealing times: (a) after annealing for 10 min, (b) after annealing for 20 min, (c) after annealing for 30 min, (d) after annealing for 40 min, (e) after annealing for 50 min, and (f) after annealing for 60 min.**


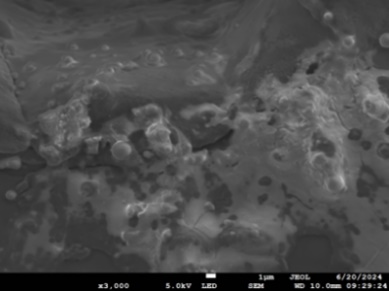

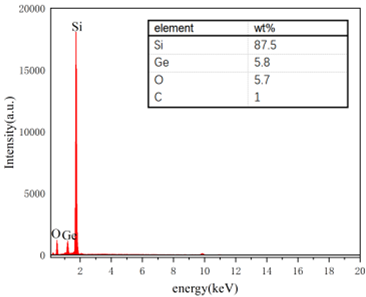


1. (b)

**S6 Fig. Characterization of the germanium-coated microcavity sample annealed for 40 min: (a) SEM image and (b) EDS spectrum.**


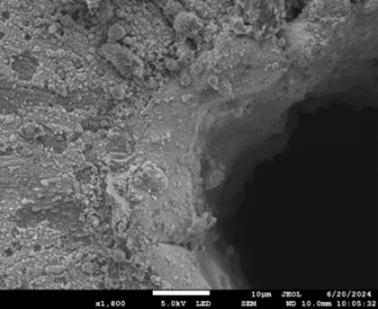

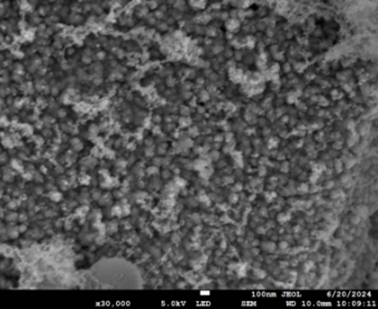


(c)

1. (b)


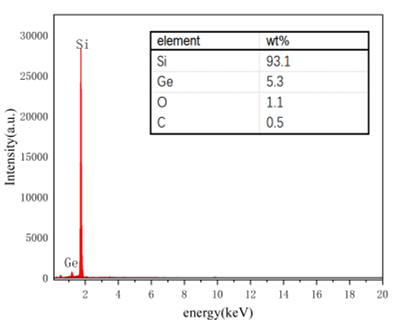


(c)

**S7 Fig. Characterization of the Ge-Si coated microcavity samples: (a) the SEM image at the edge of the microcavity, (b) an enlarged view of (a), and (c) the EDS spectrum at the edge of the microcavity.**


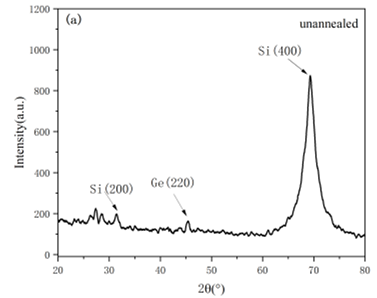

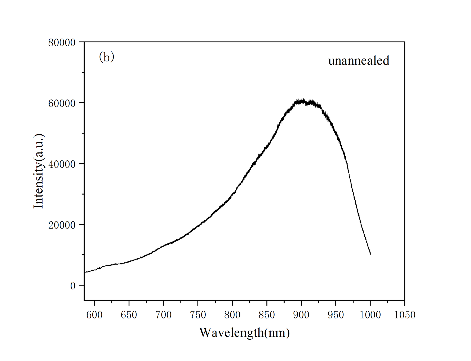


**S8 Fig. Characterization of the Ge-Si coated microcavity samples: (a) the XRD patterns, and (b) the photoluminescence spectroscopy of the samples.**


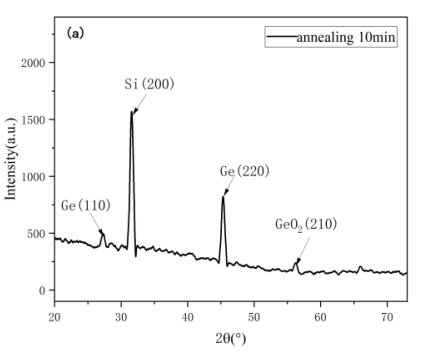

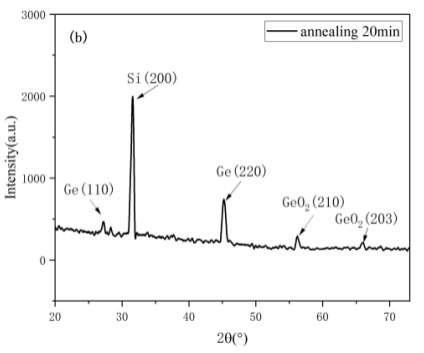

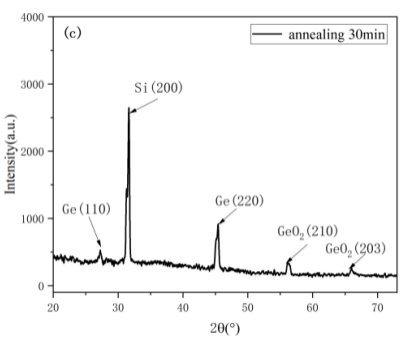

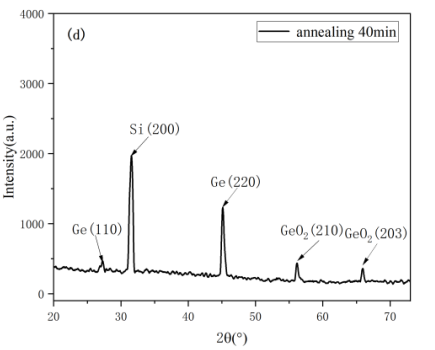

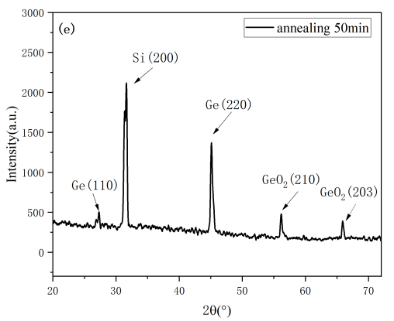

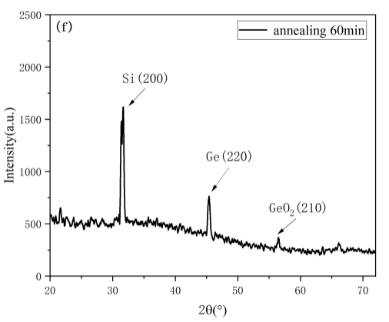


**S9 Fig.XRD patterns of Ge-Si coated microcavity samples after different annealing times: (a) XRD patterns after 10 minutes of annealing, (b) XRD patterns after 20 minutes of annealing, (c) XRD patterns after 30 minutes of annealing, (d) XRD patterns after 40 minutes of annealing, (e) XRD patterns after 50 minutes of annealing, (f) XRD patterns after 60 minutes of annealing.**


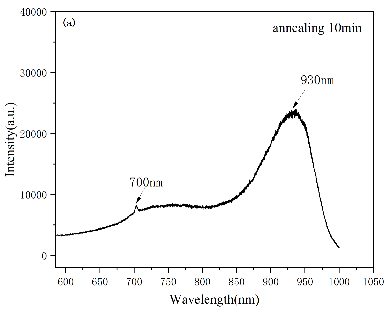

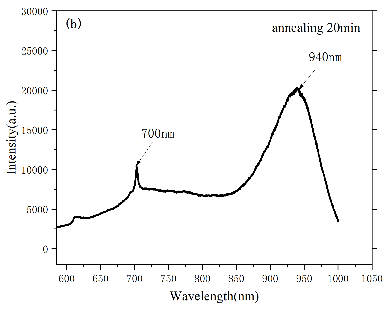

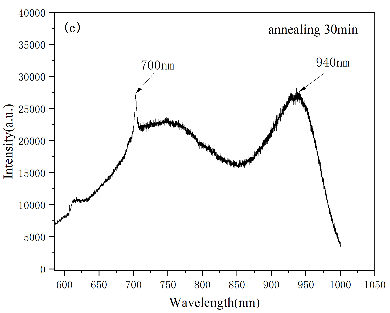

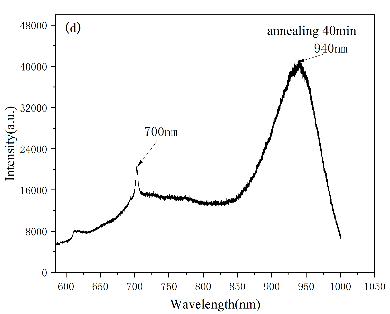

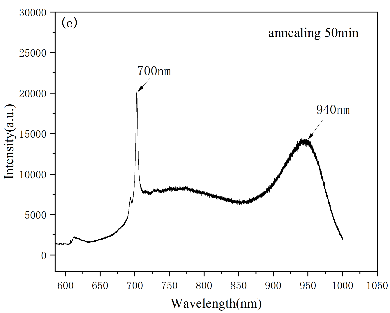

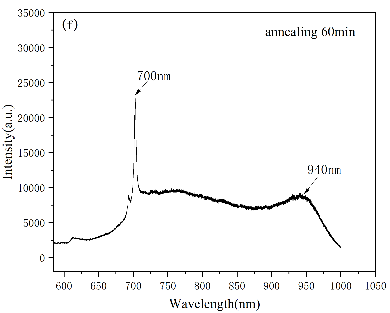


**S10 Fig. Photoluminescence spectroscopy of Ge-Si Microcavity Samples at Different Annealing Times: (a) the spectrum after 10 min; (b) after 20 min; (c) after 30 min; (d) after 40 min; (e) after 50 min; (f) after 60 min.**


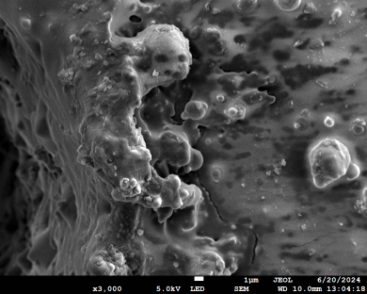

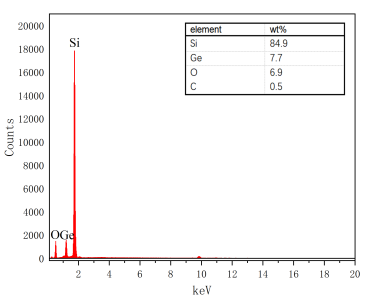


1. （b）

**S11 Fig. Characterization of the Ge-Si microcavity sample annealed for 40 min: (a) SEM image of the sample; (b) EDS image from the annealed sample.**
