# Supplementary material for: Luminescence properties of Ge and Ge-Si structures on silicon-based microcavities
Source: PLoS One. 2025 May 5;20(5):e0322559. doi: 10.1371/journal.pone.0322559 (PMC12052192; doi:10.1371/journal.pone.0322559)
Supplement: Attachment — Submitted filename: Comments R1.pdf [file pone.0322559.s003.pdf]

1) What do the authors mean when writing “Characterization images…”? Maybe, the authors could just use “characterization” in the captions?

2) The word “photoluminescence” should not be capitalized unless it begins a sentence. Please check your manuscript carefully for typos!

3)
170 “In this experiment,
171 silicon wafers were soaked in a solution of 95% concentration of
172 ethanol and acetone mixed 1:1 for five minutes to remove surface
173 organic matter, then submerged in deionized water and cleaned in an
174 ultrasonic cleaner for 15 min.”

Please, indicate the purity of the components.

4)
166 “Photoluminescence (PL) spectra were measured at
167 room”

… room temperature.

5)
205 “The deposited samples were then placed in a
206 tube furnace where they were annealed at 1000°C for varying
207 durations to study the luminescent properties of the samples with
208 different deposited elements and annealing times.”

The pressure and the atmosphere composition should be presented in the text. The pressure in the deposition chamber before the process is also absent in the text.

6)
234 “Peaks at 27° and 45° correspond to the Ge (110) and
235 Ge (220) crystal planes, respectively, and are sharp and narrow,
236 indicating high crystallinity. The peaks at 31° and 69° correspond to
237 the Si (200) and Si (400) crystal planes. Indicating the presence of
238 Ge and Si elements in the microcavity samples on the silicon
239 substrate.”

From my point of view, the statement in the text has become even less comprehensible.

The sentence written in the response to my comment is much clearer: “The XRD pattern reveals the coexistence of Ge (110) and Ge (220) crystal planes, as well as Si (200) and Si (400) crystal planes on the surface of the silicon-based microcavity. This indicates the presence of both Ge and Si elements in the microcavity samples deposited on silicon substrates.”

Sorry, I cannot understand the remark 此部分仅是回复。Google translates it as “This is a very important part of our work.”

7)
239 The 57° and 75° germanium dioxide peaks may result
240 from low peak intensities due to a small amount of oxidation on the
241 surface of the sample after preparation or during testing.

The phrase is poorly formulated. The phrase “a small amount of oxidation” is unclear.

8)
248 based clusters at the edge of the microcavity[Error! Reference source not found.].

From the authors’ response.

9) “Could the authors comment on this process by discussing what was happening to the Si, Ge and
GeO2 during annealing.

My response：As the annealing time varies, the gradual improvement in the quality of Ge-O nanocrystals
can be observed through the TO peak of XRD and Raman spectroscopy, until a certain annealing time is
reached where the Ge-O cluster structure is disrupted. The strain effect associated with annealing time is
expected to ultimately enhance the properties of the nanocrystals. The XRD peaks of both germanium
oxide nanocrystals and silicon (400) crystals dominate in the annealed samples, with the percentage of
silicon atoms decreased due to the dominance of oxygen atoms in the composition.此部分仅是回复。”

I recommend including this discussion in the manuscript.

10) “Please, clarify this paragraph regarding the origin of the line peaking at 700 nm!

My response： A group has employed a quantum dot confinement model to calculate the energy gap
structure of germanium oxide nanocrystal clusters, and utilized the Monte Carlo method to simulate the
PL spectrum and the corresponding size distribution of the germanium oxide nanocrystal clusters. The
calculated results are in agreement with our experimental findings, leading us to analyze that this is due
to the specific luminescent peaks exhibited by small-sized Ge-O clusters formed through the combination
of small-sized Ge and O.此部分仅是回复。”

In my opinion, this should be written in the text. Otherwise, the statements are quite speculative.
In addition, it is advisable to present the simulation results as a supporting material.

11) “15. Figure 6. Perhaps, oxygen came on the surface due to film oxidation in the atmosphere during
its moving to SEM. Otherwise, it is present in the SEM chamber. The treatment at 1000°C for 40
min should completely deoxidize the structure.
My response： The sample surface is populated with many small-sized germanium crystal clusters. When
the sample is processed under annealing conditions, the germanium crystals on the surface react with
oxygen atoms. After the oxygen atoms diffuse into the germanium layer, they coalesce into Ge-O clusters
above the oxide layer. However, annealing for a duration longer than a certain threshold can disrupt the
cluster structure of Ge-O. 此部分仅是回复。”

Unfortunately, the description of the annealing conditions is absent in the text. They should be presented.

12) “18. Section 3.4. The authors should explain where oxygen appears from in their experiment on
annealing. Does the deposition chamber contain a lot of O2 gas in its atmosphere? Otherwise, the
structure should deoxidize at such a high temperature. A few minutes should be enough to remove
oxygen completely from the structure in vacuum or oxygen-free residual atmosphere.

My response：The deposition process is carried out under vacuum conditions, while the annealing process
is conducted in an atmospheric environment. The source of oxygen during annealing is the oxygen
present in the atmosphere, which reacts with the surface Ge under high temperature to form a cluster
structure of Ge-O.此部分是回复.”

This should be stated in the text.
