# Supplementary material for: Luminescence properties of Ge and Ge-Si structures on silicon-based microcavities
Source: PLoS One. 2025 May 5;20(5):e0322559. doi: 10.1371/journal.pone.0322559 (PMC12052192; doi:10.1371/journal.pone.0322559)
Supplement: Attachment — Submitted filename: Response to Reviewers-20241219.docx [file pone.0322559.s004.docx]

My response to reviewers

Reviewer #1: **The micro–Raman TO broader peak seems to display it. Is there any evidence to retain its crystallinity?**

My response：Thank you for your review. As far as I know, XRD is currently used to measure crystalline properties. How to retain the crystallinity of crystals is a thought-provoking question. I'm sorry, but in my future research career, I will continue to explore it.

Reviewer #2:

1. **Section 2.1 Experimental Equipment**
   **The authors do not give information on the type and brand (series) of the X-ray spectrometer used.
   The same applies to the X-ray diffractometer.
   The series of the Renishaw spectrometers is not presented either.**

My response：The fabricated films were characterized using various techniques. First,X-ray diffrac-tion (XRD) (Rigaku Ultima-IV) with Cu Ka radiation (wavelength λ= 1.5406 A) was used todetermine the crystalline structure of the films over a 20 range of 20°to 80°. Second, a JEOLJSM-7100F scanning electron microscope was used to observe the surface morphology.Ra-man spectra were measured using a RENISHAW Raman spectrometer with a 532 nm laserover the range of 100-1300cm-1. Photoluminescence (PL) spectra were measured at room. Please refer to lines 159 to 167 of the paper for details.

**2.** **Globally. The authors do not use Raman spectroscopy, but only photoluminescence one. RENISHAW Raman spectrometers usually enable studying both Raman scattering and photoluminescence using an appropriate channel.**
**In the text, abstract and figure captions, the terms such as Raman spectroscopy, Raman spectrum or Raman spectra, etc. must be replace with photoluminescence spectroscopy, photoluminescence spectrum (spectra), etc.**

My response：The relevant content has been revised in the main text, Please refer to lines 242、284、289、299、362、407、409、414、450 of the paper for details.

**3. Pages 2 and 5. “Error! Reference source not found”**

My response：Correct references have been added to the article Please refer to lines 506 to 508 of the paper for details.

**4. Figure 1. The inscription at the scale mark on the panel (a) is too small: it is difficult to see what is written.**

My response：Figure 1 The scale has been modified，Please refer to lines 209 of the paper for details.

**5. Figures 2. The authors should indicate the mode of imaging on panels (a) and (b) or in the caption. Are the images secondary electron (SE) or backscattered electron (BSE) ones?
The authors should also reformulate the caption. Panel (c) does not show an image.
Besides, the table in the insert gives “quality percentage”. Maybe the authors mean percentage content. Is this atomic or weight percentage?
Further, the axes do not show titles and units of measurements. I can see that the energy of quantum in keV is given on the x-axis and the emission intensity is given on the y-axes (in some units, perhaps in pulse/sec/eV, as it is typical for AZtec (Oxford Instruments) EDX spectrometers).**

**The same applies to Figures 6 and 7.**

My response：The image is a secondary electron (SE), Please refer to lines 224 of the paper for details. Fig. 2 (c), Fig. 6 (b), Fig. 7 (c), Fig. 11 (b) The percentage content is the weight content percentage, and the EDX axis has been modified. Please refer to lines 226、338、366、442 of the paper for details.

**6. Figures 3, 4 and 9. The units for the 2θ axes are not shown; I can see that they are degrees, yet the authors must indicate the units of measurements for all the axes.
In addition, there are no titles and units of measurements at the y-axes.
What diffractometer did the authors use? It should be indicated in Section 2.1 as well as the X-ray source (wavelength).**
Finally, the authors did not indicate the temperature at which the luminescence was measured.

My response：The axis title and unit of measurement of the XRD plot have been revised, the diffractometer model has been indicated, and the temperature at the time of the test is 21°C (indicated in the text) Please refer to lines 178 of the paper for details. The exact model has been modified in lines 159-167.

**7. Page 3, Section 2.2, paragraph 1. The surface cleaning process, including purity of cleaning agents, should be described in more details.
Obtaining of a clean Si surface is a key process for preparing a perfect structure.
Are the authors sure that the resultant Si surface was sufficiently clean after the described treatment and did not introduce uncontrolled impurities into the prepared structures?**

**What were the conditions in the chamber when etching cavities in Si?**
My response：In this experiment, silicon wafers were immersed in a 1:1 mixture of 95% concentration ethanol and acetone for five minutes to remove surface organics. Subsequently, they were soaked in deionized water and subjected to ultrasonic cleaning to eliminate other surface impurities. The treated silicon surfaces were sufficiently clean. Immediately after treatment, the silicon wafers underwent etching to ensure no other impurities were introduced. The etching process was carried out under normal atmospheric pressure, with the room temperature maintained at 21°C. 此部分仅仅是回复。
**8. “…spectrum of the sample is displayed in Figure 1(b), featuring a characteristic luminescence peak at 700 nm attributed to the silicon-based microcavity.” What are the rest peaks in the PL spectrum? As far as I can see, the sharp peak at ~547 nm is assigned to the TO phonon in crystalline Si (Raman shift ~ 520 1/cm), isn't it? The peak at 540 nm is 2TA in c-Si (Raman shift ~300 1/cm) and that at ~563 nm should be 2TO in c-Si (Raman shift ~1040 1/cm).
Are peaks related to SiO2 seen in the spectra? The Raman scattering bands associated with vibrations of the Si–O bonds in SiO2 might appear around 560 nm (Raman shift from ~800 to ~1200 1/cm) if silicon dioxide is present in the structure [DOI: 10.2138/am.2006.2075].**

# My response：Yes, the sharp peak at 547 nanometers is attributed to the TO phonon in crystalline silicon (c-Si), the peak at 540 nanometers corresponds to 2TA in c-Si, and the peak at 563 nanometers represents 2TO in c-Si. No luminescent peaks related to silicon dioxide appear in the spectrum, which may be related to our testing location, which is at the edge of the microcavity, where small-sized clusters are sputtered by the laser. Please refer to lines 183 to187 of the paper for details.

**9. Page 4, paragraph 1. What was the gas composition of the atmosphere in the deposition chamber during the process? Was it clean enough in the chamber for the process to be conducted in correct conditions? Did the authors use some high-purity gases in the chamber? To what pressure was the chamber evacuated before the process?**

My response：During the deposition process, the deposition chamber is maintained in a vacuum state with a vacuum pressure pumped down to 5×10^-4^ Pa. No gases are introduced during the process. Prior to the experiment, the vacuum chamber of the equipment is typically maintained at a vacuum pressure of 5×10^-3^ Pa. 此部分仅是回复。
**10. Section 3.1, paragraph 1. “Figure 1(a) shows…” Figure 2(a)...**
My response：The description error has been modified in the body，Please refer to lines 219 of the paper for details.

**11. Page 5. Could the authors comment on the peaks at ~57° and ~75° in Figure 3(a) and the width of the band at 69°? If the former is GeO2, why did Ge oxidize when deposited? This should be briefly discussed in the text.**

My response：The deposition process should ideally be conducted under vacuum conditions to prevent oxidation during deposition. The peaks of germanium dioxide at 57° and 75° may result from slight oxidation on the sample surface after preparation when exposed to the atmosphere or during the testing process, leading to lower peak intensities. The crystal structure of the Si (400) plane at 69° may not be perfectly formed, exhibiting defects or disorder. Please refer to lines 239to 241 of the paper for details.

**12. “The peaks at 31° and 69° correspond to the Si (200) and Si (400) crystal planes, indicating the presence of Ge and Si elements in the microcavity samples on the silicon substrate.”
Why Si peaks indicate the presence of Ge? Please, formulate the statement clearer.**

**Page 6. “Peaks at 31° and 69° correspond to the Si (200) and Si (400) crystal faces, indicating the presence of Ge and Si elements in the microcavity samples deposited on the silicon substrate, with possible incorporation of Ge elements.”
Again, why Si peaks indicate the presence of Ge? Please, formulate the statement clearer.**
My response：My response：Understanding the statement "The peaks at 31° and 69° correspond to the Si (200) and Si (400) crystal planes" in conjunction with the previous sentence "Peaks at 27° and 45° correspond to the Ge (110) and Ge (220) crystal planes, respectively, and are sharp and narrow, indicating high crystallinity," the content on Page 6 is as follows: The XRD pattern reveals the coexistence of Ge (110) and Ge (220) crystal planes, as well as Si (200) and Si (400) crystal planes on the surface of the silicon-based microcavity. This indicates the presence of both Ge and Si elements in the microcavity samples deposited on silicon substrates.此部分仅是回复。

**13. As far as I understand from Figure 4, initially, the authors see a Si(200) peak from the substrate, Si(400) from nanocrystals, two peaks from Ge and a reflection from GeO2(210) and some undiscussed reflection. After annealing for 10 min., they see the same pattern; in 20 min., the pattern is nearly the same, yet some new undiscussed reflections appear; in 30 min., the pattern mainly remains, yet Si(200) seems to have decreased, Ge (220) and (210) increased, Si (400) increased, some unidentified reflexes disappeared, whilst some intensified.
In 40 min., a strong narrow Si(400) reflex appears (poly-Si forms?). Ge(113) appears.
In 50 min., Si(400) (poly-Si?) go on growing. GeO2 decays.
In an hour, the reflexes weaken. (Why?)
Could the authors comment on this process by discussing what was happening to the Si, Ge and GeO2 during annealing.**
My response：As the annealing time varies, the gradual improvement in the quality of Ge-O nanocrystals can be observed through the TO peak of XRD and Raman spectroscopy, until a certain annealing time is reached where the Ge-O cluster structure is disrupted. The strain effect associated with annealing time is expected to ultimately enhance the properties of the nanocrystals. The XRD peaks of both germanium oxide nanocrystals and silicon (400) crystals dominate in the annealed samples, with the percentage of silicon atoms decreased due to the dominance of oxygen atoms in the composition.此部分仅是回复。
**14. Page 7. “…but with an additional sharp peak at this wavelength.”This line is seen on panel (b).“Thus, the sharp peak at 700 nm is due to localized luminescence from Ge-O bonds within these clusters.”
This statement does not seem entirely substantiated. The authors should give a justification for it.
What the authors mean when writing about the luminescence of the Ge–O bond? Do the authors mean the Raman band? GeO2 is transparent for the 532-nm laser.
Please, clarify this paragraph regarding the origin of the line peaking at 700 nm!**
My response：A group has employed a quantum dot confinement model to calculate the energy gap structure of germanium oxide nanocrystal clusters, and utilized the Monte Carlo method to simulate the PL spectrum and the corresponding size distribution of the germanium oxide nanocrystal clusters. The calculated results are in agreement with our experimental findings, leading us to analyze that this is due to the specific luminescent peaks exhibited by small-sized Ge-O clusters formed through the combination of small-sized Ge and O.此部分仅是回复。

**15. Figure 6. Perhaps, oxygen came on the surface due to film oxidation in the atmosphere during its moving to SEM. Otherwise, it is present in the SEM chamber. The treatment at 1000°C for 40 min should completely deoxidize the structure.**
My response：The sample surface is populated with many small-sized germanium crystal clusters. When the sample is processed under annealing conditions, the germanium crystals on the surface react with oxygen atoms. After the oxygen atoms diffuse into the germanium layer, they coalesce into Ge-O clusters above the oxide layer. However, annealing for a duration longer than a certain threshold can disrupt the cluster structure of Ge-O.此部分仅是回复。

**16. Figure 8. What is unannealing? Unannealed.**
My response：Figure 8 (a) and (b) have been revised，Please refer to lines 372 of the paper for details.
**17. Section 3.3. “For the prepared germanium-coated silicon-based microcavity samples, PLD was used to deposit silicon onto a silicon target, followed by a layer of silicon structured over the germanium, creating a Ge-Si bilayer structure on the microcavity samples.”
The phrase is unclear. Please, reformulate it.**
My response：The description in the text has been revised，Please refer to lines 345 to 351 of the paper for details.
**18. Section 3.4. The authors should explain where oxygen appears from in their experiment on annealing. Does the deposition chamber contain a lot of O2 gas in its atmosphere? Otherwise, the structure should deoxidize at such a high temperature. A few minutes should be enough to remove oxygen completely from the structure in vacuum or oxygen-free residual atmosphere.**

My response：The deposition process is carried out under vacuum conditions, while the annealing process is conducted in an atmospheric environment. The source of oxygen during annealing is the oxygen present in the atmosphere, which reacts with the surface Ge under high temperature to form a cluster structure of Ge-O.此部分是回复.
