# Supplementary material for: Luminescence properties of Ge and Ge-Si structures on silicon-based microcavities
Source: PLoS One. 2025 May 5;20(5):e0322559. doi: 10.1371/journal.pone.0322559 (PMC12052192; doi:10.1371/journal.pone.0322559)
Supplement: Attachment — Submitted filename: My Response to Reviewers-20250207.docx [file pone.0322559.s005.docx]

My Response to Reviewers

PLOS ONE, PONE-D-24-46249R1

Luminescence Properties of Ge and Ge-Si Structures on Silicon-Based Microcavities

1. Additional Editor Comments:
The manuscript needs a thorough revision to address several issues related to typos, missing words, proper capitalization, and sentence structure for better clarity and readability.
Specific comments for figures and presentation:
General formatting: Ensure all graphs have the same font size and avoid using bold fonts in SEM images. Use a white background for all figures. Remove unnecessary boxes around titles (e.g., "unannealed" or "annealing at different timings").

My response：I have made the corresponding modifications, please refer to lines 232、361、389、and 470 for details.
Figure-specific Comments:
Figure 2c: Missing information for the x-axis (Energy in KeV) and y-axis (Intensity(a.u.)).

My response：I have made the corresponding modifications, please refer to lines 233 for details.
Figure 3a & 3b: Y-axis information is missing for Figure 3a. the font style differs between Figures 3a and 3b when compared to Figure 2. Maintain uniformity.

My response：I have made the corresponding modifications, please refer to lines 255 for details.
Figure 4: Clearly label the y-axis (e.g., Intensity (a.u.)). The inset in figure 4e is unclear and needs to be replotted for better visibility.

My response：I have made the corresponding modifications, please refer to lines 282、283 and 284 for details.
Figure 5a: The line thickness is inconsistent compared to other lines in the figure. Ensure uniformity.

My response：I have made the corresponding modifications, please refer to lines 343 for details.
Figures 6a, 6b, and 6c: Replace bold fonts with normal fonts and use a white background.

My response：I have made the corresponding modifications, please refer to lines 361 for details.
Figure 7: Use normal font for labels (a, b, c). X-axis and y-axis information is missing.

My response：I have made the corresponding modifications, please refer to lines 389 and 390 for details.
Figure 8a: Y-axis information is missing.

My response：I have made the corresponding modifications, please refer to lines 394 for details.
Figure 9: Y-axis information is missing in all parts.

My response：I have made the corresponding modifications, please refer to lines 423、424 and 425 for details.
Figure 11: Revise this figure to match the updated standards.

My response：I have made the corresponding modifications, please refer to lines 470 for details.
Axis Intervals: Across Figures 1 to 11, the y-axis has too many interval points. Use larger intervals to make the data presentation clearer.

My response：I have made the corresponding modifications, please refer to lines 233 255 282 283 284 343 344 345 361 390 394 423 424 425 452 453 454 and 470 for details.

2. Reviewer Questions:


Reviewer #1: The manuscript wording has been revised. Anyway, the Raman spectroscopy is not photoluminescence. Please address the real methodology and marked it properly. The high spectral resolution of a Raman spectrometer can be useful for PL spectroscopy, especially when the emission spectra have narrow bands or lines. Though it is not mentioned properly.

My response：The text has been revised——In this study, a Renishaw Raman spectrometer was used to test the PL spectroscopy of the sample, which had an excitation light of 532 nm, a spectral detection range of 547-900 nm, and an excitation power of up to 50 mW； “Please refer to lines 167 to 170 of the paper for details”.

Reviewer #2: 1) What do the authors mean when writing “Characterization images…”? Maybe, the authors could just use “characterization” in the captions?
My response：The characterization images have been modified to 'characterization' in the text.“Please refer to lines 216、256、362、391、395、471 of the paper for details”.

2) The word “photoluminescence” should not be capitalized unless it begins a sentence.
Please check your manuscript carefully for typos!
My response：Change some photoluminescence that is not at the beginning to lowercase letters.“Please refer to lines 185、217、247、257、289、294、310、385、396、429、431、436、478 and 543 of the paper for details”.

3)
170 “In this experiment,
171 silicon wafers were soaked in a solution of 95% concentration of
172 ethanol and acetone mixed 1:1 for five minutes to remove surface
173 organic matter, then submerged in deionized water and cleaned in an
174 ultrasonic cleaner for 15 min.”

Please, indicate the purity of the components.
My response：The text has been revised—In this experiment, silicon wafers were immersed in a mixed solution of 30 milliliters of 95% concentrated ethanol and 30 milliliters of 98% concentrated acetone for 5 minutes to remove surface organics. Afterward, the silicon wafers were cleaned with deionized water using an ultrasonic cleaner for 15 minutes；“Please refer to lines 173 to 178 of the paper for details”.

4)
166 “Photoluminescence (PL) spectra were measured at
167 room”

… room temperature.
My response：Room temperature has been added to the main text. Please refer to lines 51of the paper for details.

5)
205 “The deposited samples were then placed in a
206 tube furnace where they were annealed at 1000°C for varying
207 durations to study the luminescent properties of the samples with
208 different deposited elements and annealing times.”

The pressure and the atmosphere composition should be presented in the text. The pressure in the deposition chamber before the process is also absent in the text.
My response：Add annealing conditions. Please refer to lines 209 to 214 of the paper for details.

6)
234 “Peaks at 27° and 45° correspond to the Ge (110) and
235 Ge (220) crystal planes, respectively, and are sharp and narrow,
236 indicating high crystallinity. The peaks at 31° and 69° correspond to
237 the Si (200) and Si (400) crystal planes. Indicating the presence of
238 Ge and Si elements in the microcavity samples on the silicon
239 substrate.”

From my point of view, the statement in the text has become even less comprehensible.
The sentence written in the response to my comment is much clearer: “The XRD pattern reveals the coexistence of Ge (110) and Ge (220) crystal planes, as well as Si (200) and Si (400) crystal planes on the surface of the silicon-based microcavity. This indicates the presence of both Ge and Si elements in the microcavity samples deposited on silicon substrates.”
Sorry, I cannot understand the remark. Google translates it as “This is a very important part of our work.”
My response：The text has been revised—The XRD pattern reveals the coexistence of Ge (110) and Ge (220) crystal planes, as well as Si (200) and Si (400) crystal planes on the surface of the silicon-based microcavity； Please refer to lines 240 to 242 of the paper for details.

7)
239 The 57° and 75° germanium dioxide peaks may result
240 from low peak intensities due to a small amount of oxidation on the
241 surface of the sample after preparation or during testing.

The phrase is poorly formulated. The phrase “a small amount of oxidation” is unclear.
My response：The text has been revised—In the XRD pattern, there are also two low peaks located at 57° and 75° , indicating the generation of weak germanium dioxide crystal phase during laser etching； Please refer to lines 243 to 245 of the paper for details.

8)
248 based clusters at the edge of the microcavity Error! Reference source not found..


From the authors’ response.

My response：Add reference information. Please refer to lines 252 of the paper for details.

9) “Could the authors comment on this process by discussing what was happening to the Si, Ge and GeO2 during annealing.
My response：As the annealing time varies, the gradual improvement in the quality of Ge-O nanocrystals can be observed through the TO peak of XRD and Raman spectroscopy, until a certain annealing time is reached where the Ge-O cluster structure is disrupted. The strain effect associated with annealing time is expected to ultimately enhance the properties of the nanocrystals. The XRD peaks of both germanium oxide nanocrystals and silicon (400) crystals dominate in the annealed samples, with the percentage of silicon atoms decreased due to the dominance of oxygen atoms in the composition.
I recommend including this discussion in the manuscript.
My response：It has been added in the body—As the annealing time changes, it can be observed through XRD and Raman spectroscopy that the TO peak of Ge-O gradually increases, and the crystallization performance improves until a certain annealing time is reached. The Ge-O cluster structure is destroyed, and the crystallization performance deteriorates. In the annealed samples, XRD peaks of germanium oxide nanocrystals and silicon (400) crystals dominate, and the percentage of silicon atoms decreases due to the dominant position of oxygen atoms in the composition; Please refer to lines 318 to 326 of the paper for details.

10) “Please, clarify this paragraph regarding the origin of the line peaking at 700 nm!
My response：A group has employed a quantum dot confinement model to calculate the energy gap structure of germanium oxide nanocrystal clusters, and utilized the Monte Carlo method to simulate the PL spectrum and the corresponding size distribution of the germanium oxide nanocrystal clusters. The calculated results are in agreement with our experimental findings, leading us to analyze that this is due to the specific luminescent peaks exhibited by small-sized Ge-O clusters formed through the combination of small-sized Ge and O.”
In my opinion, this should be written in the text. Otherwise, the statements are quite speculative.
In addition, it is advisable to present the simulation results as a supporting material.

My response：It has been added in the body—Huang W Q had employed a quantum dot confinement model to calculate the energy gap structure of germanium oxide nanocrystal clusters, and utilized the Monte Carlo method to simulate the PL spectrum and the corresponding size distribution of the germanium oxide nanocrystal clusters. The calculated results are in agreement with our experimental findings; Please refer to lines 302 to 307 of the paper for details; Reference added on line 579, line 581.

11) “15. Figure 6. Perhaps, oxygen came on the surface due to film oxidation in the atmosphere during its moving to SEM. Otherwise, it is present in the SEM chamber. The treatment at 1000°C for 40 min should completely deoxidize the structure.
My response：The sample surface is populated with many small-sized germanium crystal clusters. When the sample is processed under annealing conditions, the germanium crystals on the surface react with oxygen atoms. After the oxygen atoms diffuse into the germanium layer, they coalesce into Ge-O clusters above the oxide layer. However, annealing for a duration longer than a certain threshold can disrupt the cluster structure of Ge-O.
Unfortunately, the description of the annealing conditions is absent in the text. They should be presented.
My response：It has been added in the body—The sample surface is populated with many small-sized germanium crystal clusters. When the sample is processed under annealing conditions, the germanium crystals on the surface react with oxygen atoms. After the oxygen atoms diffuse into the germanium layer, they coalesce into Ge-O clusters above the oxide layer. However, annealing for a duration longer than a certain threshold can disrupt the cluster structure of Ge-O；Please refer to lines 331 to 337 of the paper for details.

12) “18. Section 3.4. The authors should explain where oxygen appears from in their experiment on annealing. Does the deposition chamber contain a lot of O2 gas in its atmosphere? Otherwise, the structure should deoxidize at such a high temperature. A few minutes should be enough to remove oxygen completely from the structure in vacuum or oxygen-free residual atmosphere.
My response：The deposition process is carried out under vacuum conditions, while the annealing process is conducted in an atmospheric environment. The source of oxygen during annealing is the oxygen present in the atmosphere, which reacts with the surface Ge under high temperature to form a cluster structure of Ge-O.

This should be stated in the text.

My response：It has been added in the body—The source of oxygen during annealing is the oxygen present in the atmosphere, which reacts with the surface Ge under high temperature to form a cluster structure of Ge-O；Please refer to lines 444 to 447 of the paper for details.
