# Supplementary material for: Luminescence properties of Ge and Ge-Si structures on silicon-based microcavities
Source: PLoS One. 2025 May 5;20(5):e0322559. doi: 10.1371/journal.pone.0322559 (PMC12052192; doi:10.1371/journal.pone.0322559)
Supplement: Attachment — Submitted filename: renamed_487c4.docx [file pone.0322559.s006.docx]

My Response to Reviewers

We've checked your submission and before we can proceed, we need you to address the following issues:

1. Thank you for stating the following financial disclosure:
[This work was supported by the National Natural Science Foundation of China (U1704145), the Hainan Provincial Natural Science Foundation of China (522MS062), and the specific research fund of the Innovation Platform for Academicians of Hainan Province (YSPTZX202207).].

Please state what role the funders took in the study. If the funders had no role, please state: "The funders had no role in study design, data collection and analysis, decision to publish, or preparation of the manuscript."
If this statement is not correct you must amend it as needed.
Please include this amended Role of Funder statement in your cover letter; we will change the online submission form on your behalf.
2. Please note that funding information should not appear in any section or other areas of your manuscript. We will only publish funding information present in the Funding Statement section of the online submission form. Please remove any funding-related text from the manuscript.

My response：We have removed the acknowledgments content and added the phrase 'Professor Zhao Shihua, the sponsor, the corresponding author, played a role in research design, data analysis, publication decision, and manuscript revision in this paper' to the system.

3. Please include the captions for the Supporting Information files at the end of your manuscript.

My response：Thank you for your reply. The funding information in the manuscript has been removed, and the modifications are reflected in lines 491 of the text. Relevant title information for the supporting information files has been added at the end of the manuscript, and the changes are embodied in lines 575 to 609 of the document.
